# Postadychute-AG, Detection, and Prevention of the Risk of Falling Among Elderly People in Nursing Homes: Protocol of a Multicentre and Prospective Intervention Study

**DOI:** 10.3389/fdgth.2020.604552

**Published:** 2021-01-27

**Authors:** Flavien Quijoux, François Bertin-Hugault, Philippe Zawieja, Marie Lefèvre, Pierre-Paul Vidal, Damien Ricard

**Affiliations:** ^1^Centre Borelli UMR 9010/Université Paris-Saclay, ENS Paris-Saclay, CNRS, SSA, Université de Paris, Inserm, Paris, France; ^2^ORPEA Group, Puteaux, France; ^3^Institute of Information and Control, Hangzhou Dianzi University, Hangzhou, China; ^4^Service de Neurologie de l'Hôpital d'Instruction des Armées de Percy, Service de Santé des Armées, Clamart, France; ^5^Ecole du Val-de-Grâce, Ecole de Santé des Armées, Paris, France

**Keywords:** prediction, fall, elderly people, physical activity, balance quantification

## Abstract

**Introduction:** While falls among the elderly is a public health issue, because of the social, medical, and economic burden they represent, the tools to predict falls are limited. Posturography has been developed to distinguish fallers from non-fallers, however, there is too little data to show how predictions change as older adults' physical abilities improve. The Postadychute-AG clinical trial aims to evaluate the evolution of posturographic parameters in relation to the improvement of balance through adapted physical activity (APA) programs.

**Methods:** In this prospective, multicentre clinical trial, institutionalized seniors over 65 years of age will be followed for a period of 6 months through computer-assisted posturography and automatic gait analysis. During the entire duration of the follow-up, they will benefit from a monthly measurement of their postural and locomotion capacities through a recording of their static balance and gait thanks to a software developed for this purpose. The data gathered will be correlated with the daily record of falls in the institution. Static and dynamic balance measurements aim to extract biomechanical markers and compare them with functional assessments of motor skills (Berg Balance Scale and Mini Motor Test), expecting their superiority in predicting the number of falls. Participants will be followed for 3 months without APA and 3 months with APA in homogeneous group exercises. An analysis of variance will evaluate the variability of monthly measures of balance in order to record the minimum clinically detectable change (MDC) as participants improve their physical condition through APA.

**Discussion:** Previous studies have stated the MDC through repeated measurements of balance but, to our knowledge, none appear to have implemented monthly measurements of balance and gait. Combined with a reliable measure of the number of falls per person, motor capacities and other precipitating factors, this study aims to provide biomechanical markers predictive of fall risk with their sensitivity to improvement in clinical status over the medium term. This trial could provide the basis for posturographic and gait variable values for these elderly people and provide a solution to distinguish those most at risk to be implemented in current practice in nursing homes.

**Trial Registration:** ID-RCB 2017-A02545-48.

**Protocol Version:** Version 4.2 dated January 8, 2020.

## 1. Introduction

Elderly falls are defined by the World Health Organization as “an event in which a person [over the age of 65] inadvertently falls to a lower level on the ground or any other surface than where he or she was previously” (1). But behind this simple definition, the falls of frail people bring together many economic and social issues. The incidence of falls among people over 65 years old is estimated at 30% (2). With the risk of falling increasing with age, 24% of people over 80 years old fall recurrently (i.e., at least twice a year) (3). It was estimated in 2004 in France that 450,000 accidents requiring a visit to hospital emergency departments were linked to falls, making it the most common accident in everyday life. Among these accidents, there were nearly 9,000 fatal falls of elderly people (4). Although the percentage of fatal falls remains low, the consequences are both frequent and disabling. The main factors identified by the French Health Authority (5) are (1) the age of the person, (2) the institutionalization (39% of the residents of retirement homes had at least two falls in 1 year), and (3) the polymorbidity, although this factor is not specific, is an important risk factor. Institutionalized people over 75 years of age have the highest fall rates with 0.6 to 3.6 falls per person per year (6, 7). Frailty, defined as a precarious state where physiological and social factors may increase an existing functional loss (8), must be taken into account to prevent falls. The frailty of elderly people should be measured through the following indicators: weight loss, self-reported exhaustion, physical weakness, slow walking speed, and low physical activity. Although screening for these risk factors is common in geriatric care, the analysis of these factors alone does not provide sufficient sensitivity and specificity to identify individuals at high risk of falling (9). Given these limitations, exhaustive analysis of the fall risk factors has only a limited value, and in practice, a questionnaire on the history of falls and the expertise of the professional are more often preferred to identify people who will eventually fall in the coming months (10).

To quantify the risk of falling in older population, static posturography can then be used (11–14). It examines the orientation and body movements during a quiet stance task through a force platform and the computation of the center of pressure (COP) trajectory. Still, this recording remains an examination reserved to specialists of human biomechanics (15, 16). Indeed, this method could not be applied in current practice mainly because of its ergonomics, cost, and the difficulty of interpreting the results. On the other hand, the development of portable force platforms, such as the Wii Balance Board (WBB) [Nintendo, Tokyo, Japan], has democratized the use of static posturography. The reliability of the measurements made with the WBB has been studied extensively (17–23). With the WBB, it is possible to measure the total sway path length, the mean velocity of the COP, the sway area enclosing the COP displacement, and other parameters classically used for postural control analysis (14, 24–26). However, their statistical interpretation is usually time consuming and incompatible with clinical practice. In addition, the number of parameters derived from the COP trajectory can quickly exceed several dozen and the experimental recording conditions vary greatly between studies (27). Determining which parameters in which experimental conditions are optimal to discriminate future fallers from elderly non-faller remains a difficult task (28–30).

Similarly, it can be interesting to look for predictive markers in the gait analysis, since falling is also correlated with gait disorders (31–33). Thus, by adding inertial measurement units (IMU) on different segments of the body, it is possible to study the spatiotemporal alteration of the gait due to aging or pathologies through inertialocography (ILG) (34, 35) Biomechanics parameters in gait analysis (such as gait speed, number of steps for a U turn, average duration of a step, and so on) allow an early identification of future fallers (36, 37). However, while these two methods of analysis of static and dynamic balance, posturography and ILG, respectively, provide clues for the early detection of elderly people at risk of falling, few studies combine both (38–41). Once identified, people at high risk of falling could then benefit from an optimized and individualized follow-up. Yet, few studies have quantified the variability of biomechanical parameters during longitudinal monitoring, although these data are essential to demonstrate a deviation from the normal evolution of these parameters. This study aims at determining the evolution of the computed predictive markers to physical capacities improvement in elderly people living in nursing home.

Concerning the risk of falling, adapted physical activity (APA) has shown its benefits (42, 43). In addition to reducing the number of falls in the elderly (44–47), APA has shown an improvement in clinical and functional tests (48, 49) as well as in biomechanical markers related to falls in static (50) and dynamic balance (51). Although there is no consensus on the definition of APA, particularly with regard to sports practices or functional tasks included in daily life activities (52), adapted exercises include physical activities of various intensities and generally multi-component. According to the 2008 Physical Activity Guidelines for Americans (53), “*exercise is a form of physical activity that is planned, structured, repetitive, and performed with the goal of improving health or fitness*.” Physical exercises can be proposed in order to improve predictive markers, gait quality, and finally functional test scores. Therefore, it would be appropriate to provide older persons with the right rehabilitation program to improve their balancing abilities. Non-medication and exercise-based interventions have indeed shown significant effects on the number of falls and associated trauma (54), quantified by a median absolute reduction of 3 falls per participant and a lower rate of traumatic falls by a median reduction of 0.35 traumatic falls per person-year (55). The American Geriatrics Society (46) reported a positive impact of APA, in general, on the physical capacities of the elderly without noting any correlation between the duration or intensity of the efforts and the expected benefits. This difficulty in determining the best program for each individual is also retained by the Cochrane Collaboration, which indicates in its meta-analysis a mitigated effect of interventions to reduce the risk of falls for nursing home residents (44). Hence, despite the recommendations regarding APA for elderly people (56), no optimal APA program seems to be clearly described, especially for nursing home residents (44). In addition, the impact of APA on motor capacities of people with mild-to-severe cognitive impairments remains a matter of debate (57). This highlights the challenge of optimizing the follow-up of elderly people following physical exercise programs.

## 2. Method and Design

### 2.1. Aim and Objectives

As part of a wider project aiming to develop a numeric tool for individual longitudinal monitoring using quantitative approaches (58), the objective of the Postadychute-AG clinical trial is to validate the reliability of predictive models previously published (59–63) on prospective follow-up of institutionalized older adults and to quantify their sensitivity to physical rehabilitation. In order to achieve this objective, the risk of falls predicted each month by means of instrumental assessment of balance and gait, respectively, via posturography and ILG, will be compared with the number of falls actually recorded during that month by the nursing home's medical staff. Falls will be recorded daily using a standardized form.

We hypothesize that a score based on both evaluations of static and dynamic balance allows a reliable and unequaled estimation of the risk of falling, after selection of the best biomechanics parameters. This selection is based on systematic reviews (30, 35, 64), machine learning algorithms from previous recordings (11, 12), and on an analysis of locomotion made in consultation for patients with or without neurological disorders (65).

A previous prospective study has shown the value of combining static and dynamic balance analysis to predict the risk of falling in the elderly (66). However, limited data are available for institutionalized people. On the other hand, an improvement in the quality of static balance and gait can be expected after 3 months of physical training (67, 68). The sensitivity of predictive models to the improvement of physical abilities (or their deterioration) remains unknown. Hence, this represents a major challenge to guide rehabilitation and optimize care in order to reduce the number of falls and their consequences (69).

The secondary objective of the Postadychute-AG clinical trial is to evaluate the acceptability of the measurement method by the healthcare personals of the selected nursing homes. If the results of the study prove to be relevant and the instrumental assessment method is well-received by carers, the ultimate goal is to eventually recommend the use of these balance and gait measurements to assist professionals in the diagnosis and management of balance disorders.

### 2.2. Trial Design

This study is a low-risk and low-constraint multicenter clinical trial following prospectively a cohort of older people (≥ 65 years old) in 16 institutions. Each nursing home will enroll 5–15 participants for 6 months, then renew the list of participants at the end of the 6-month follow-up. All the selected residents from the 16 nursing homes will be included in an APA program after 3 months of follow-up. Hence, they will be monitored during 3 months without APA and then 3 more months with APA. Four different APA programs are planned. One or two APA programs, among the four possible, are then chosen by the healthcare team and can be changed after the 6-month follow-up. The comparisons will be intra-individuals in the different programs, i.e., between pre-APA and post-APA evaluations in a pairwise manner.

To the extent that APA reduces the risk of falling in institutionalized elderly patients (70), we want to test the robustness of the developed predictive models on the evolution of the risk of falling. The objective is to evaluate the sensitivity of models using static and dynamic balance predictive markers. The first 3 months of follow-up without APA will thus serve as a baseline to quantify the evolution of the participants' balance. The 3 months with APA will allow to quantify the improvement in balance abilities. In both phases of the trial (without and with APA), the predictive falls risk score will be correlated with the daily recording of falls.

In order to propose physical activity that is most adapted to the abilities of the people included in the study, 4 groups will be defined as follows:

- (1) The first physical activity group will be designed for older people with a low risk of falling;
- (2) Older people at high risk of falling;
- (3) Those with a high risk of falling but also with cardiovascular problems that necessarily require a adjustment of the physical exercises program;
- (4) The fourth group will consist of people with cognitive impairment that is incompatible with similar care to the other groups, given the deficiencies and attention disorders associated with their mild-to-severe cognitive impairments.

In each institution, one or two physical activity groups will be constituted until the renewing of the list of participants (every 6 months). The total duration of the study is 24 months.

#### 2.2.1. Measurements and Outcomes

The instrumented measurement of balance and gait will need to be related to clinical measurements of motor skills such as the Berg Balance Scale (BBS) (71) or Mini Motor test (MMT) (72) in order to provide relevant information on the motor strategies of older people who fall (73).

In the normal course of resident care, the motor function assessment is already done clinically using functional scales such as the BBS. For more fragile residents, the MMT can be preferred. Both provide a numerical score of the balance capacities. The BBS, or the MMT when more appropriate, will be performed at the inclusion of the resident in the trial. In addition to these two scales, the assessment of the cognitive state of the residents is carried out with the Mini Mental State Examination (MMSE). A pain assessment will also be performed through Algoplus (74), Doloplus (75), or at least quoted from 0 to 10 with the localization indicated. The three scales provide a score to quantify the pain. In case of variability in the use of the different scales, their transformation into a normalized effect size will be calculated. The Autonomy, Gerontology Group Iso Resources (AGGIR) grid (76) is a categorical score to determine the level of dependency of the participants. The AGGIR grid evaluates the dependency of the elderly people by scoring from GIR 1 (presence of continuous human assistance required) to GIR 6 (fully autonomous) the aids needed to perform daily tasks. This grid, as well as the BBS, the MMT, the MMSE, and the pain scales are used clinically on a very frequent basis by the care providers in nursing home. The correlation of biomechanical markers with the clinical evaluations could provide a better understanding of the markers evolution to the healthcare professionals.

In addition to these measures, a systematic search for the main risk factors for falls and cardiovascular disease will be carried out using an assessment form developed for this clinical trials (ORPEA Risk Assessment Sheet [ORAS]). The ORAS form is an assessment of the elderly person's fall and cardiovascular risks for inclusion in the four APA programs planned in Postadychute-AG. It is filled in by healthcare professionals (coordinating physician of the nursing home and physiotherapists). It consists mainly of check boxes. The ORAS form gathers factors predisposing to falls, a neuropsychiatric evaluation, an evaluation of cardiovascular and pulmonary disorders, a stress test adapted to institutionalized elderly people, a frailty questionnaire, a functional measurement of sitting, standing balance and mobility, as well as an evaluation of cognitive abilities. Additional comments and information may be added by the healthcare professional, specifying possible contraindications to the APA that have not been mentioned in the form, if any (see Additional File 3 for more information). The ORAS assessment contains an endurance exercise that aims to assess the feasibility of the participant to sustain continuous physical activity for several minutes. During this stress test, the person is monitored with an electronic tensiometer and a saturometer to check the evolution of blood pressure, heart rate, and oxygen saturation. The same cardiovascular assessment is carried out in the first APA sessions to check the adequacy of the intensity of the exercises with the physical capacities of the participants.

**Figure 1:**
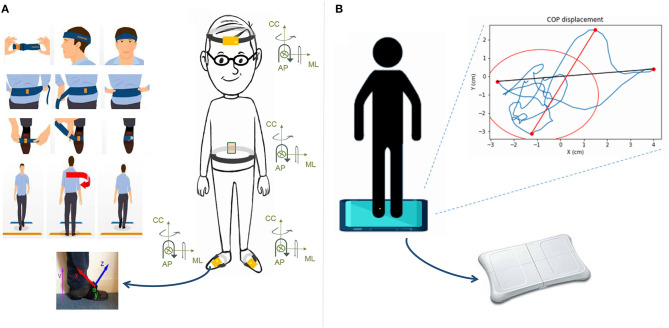
Measurement process for balance analysis. **(A)** For gait analysis: Four sensors are placed on the top of each foot, on the low back, and on the front of the resident.They are asked to walk 10 m, make a U-turn and come back to the start line. The orientation of each IMU is indicated. **(B)** For quiet stance analysis: The force platform is a Wii Balance Board on which the participant stands and remains as still as possible for two periods of 25 s (eyes open and eyes closed). The signal of the COP trajectory is then recorded and automatically analyzed.

Both recordings (static and dynamics balance measures) will therefore be made monthly for 6 months (Figure 1). The first one is a recording of their static balance standing on a WBB, 25-s eyes open and then 25-s eyes closed, arms along the body. The position of the feet will be wide enough to be comfortable, not exceeding the width of the shoulders. The posturographic data are transmitted to a tablet and presented to the healthcare professional, thanks to a software specially developed for this purpose, with multiple visualizations and a summary of the main COP parameters. The other recording is a 10-m gait exercise both ways with IMU on the feet, lumbar region, and head following this sequence: (1) standing still for 6 s, (2) walking 10 m at the speed chosen to be comfortable, (3) U-turn, (4) walking 10 m at the same speed, and (5) stopping and standing still for 3 s. In both cases, the data are transmitted via Bluetooth to the tablet held by the healthcare professional. Within seconds, the signals are pre-processed (77, 78) and shown to the healthcare professional for interpretation.

The recording of these large amounts of data, and their correlation, should make it possible to analyze the various factors that can lead to a fall in the elderly person (79).

### 2.3. Study Settings and Participants

#### 2.3.1. Regulatory Submission Procedures

This protocol is in conformity with the internationally recognized ethical principles of the Declaration of (80) and its amendments. The protocol, its summary, the information note and the consent form for the study, accompanied by other administrative papers, have been submitted for authorization to the Protection of Persons Committee (CPP) chosen randomly in accordance with the laws in force (authorization dated June 14, 2019). The CPP Nord Ouest IV has validated the safety and methodology of this protocol (National number: 2017 A02545 48, dated June 14, 2019). The protocol is also registered with ANSM under the number 2017-A02545-48. Amendments to the protocol leading to significant changes or modifying the constraints or risks incurred by the participants will first be submitted to the CPP for validation. The data collection and the protection measures have been validated by the National Commission for Information Technology and Civil Liberties (CNIL) (authorization dated December 6, 2019). The results of the study will be exploited after pseudonymization and in an aggregated manner.

#### 2.3.2. Eligibility Criteria

For this study, the participants will have to meet the following inclusion criteria:

- Adults of both sexes, aged 65 years and over, social insurance recipients;
- Living in one of the selected nursing home of the ORPEA private group;
- Not presenting any neurological, vestibular, or visual disorder incompatible with stepping on the WBB platform or walking 10 m without human assistance;
- Being able to safely stepping on the WBB platform (as estimated by the investigating practitioner) and able to maintain a quiet stance for more than 1 min, with eyes open or closed;
- Have obtained an MMSE score higher than 18 on the date of entry into the nursing home or within the last 6 months;
- Having an assumed life expectancy of more than 6 months, as estimated by the coordinating physician of the establishment;
- Having signed the informed consent.

The criteria that do not allow to be included in the study gather (1) non-mobile residents (any person with a musculoskeletal or neurosensory disorder that does not allow him/her to maintain a standing position for more than 1 min on the WBB force platform), (2) history of limb amputation, (3) blindness (assessed using an Amsler grid), or (4) the resident refusal.

The resident can be discharged from the trial by choice or due to an inability to continue static and dynamic assessments (e.g., related to a serious adverse event due to a fall for example). An intention-to-treat analysis will be applied and data from participants who do not complete the protocol will be used until the date of their exclusion from the trial or their death.

#### 2.3.3. Recruitment

Individuals will be recruited from their nursing home in Paris and the Île-de-France region in France. This community will therefore remain relatively homogeneous despite possible differences, particularly with regard to cognitive disorders that may require residence in a protected unit. The cognitive deficits, fall risks, and cardiovascular risks presented by the participants lead to the following allocation in the four APA programs:

- Group 1 “Maintaining Autonomy”: Participants of group 1 have no cardiovascular anomaly detected during the stress test, but possibly some limited cognitive deficits (MMSE > 24 and cognitive abilities considered as sufficient through the ORAS form; Additional File 3). They do not live in a protected unit. They represent a group of robust residents, with limited risk of falling, living autonomously in their rooms and inside the nursing home with or without walking aids. The goal of this first group is to prevent additional risk factors and reduce the consequences of falls.
- Group 2 “Prevention of the risk of falling”: Participants of group 2 have no cardiovascular anomaly detected during the stress test, but possibly some limited cognitive deficits. They possibly can walk alone in the nursing home but still present a significant risk of falling (with a history of recurrent falls in the last 6 months). Patients in wheelchairs can be included in this group if they are able to do stand up on demand. The goal of this second APA program is to improve motor abilities and balance.
- Group 3 “Prevention of the risk of falling and monitoring of cardiovascular disorders in the absence of cognitive impairment”: Group 3 participants have had a recent but stabilized cardiovascular disorder or an abnormality detected during the stress test. These disorders require specific management. Participants in wheelchairs can be included in the group 3. The objective of group 3 is to manage to reduce the risk of falling by adapting the exercises to the cardiovascular capacities, thus improving endurance for better autonomy.
- Group 4 “Prevention of the risk of falls in a context of cognitive deficit”: Participants in group 4 present moderate to severe cognitive deficits. Cognitive impairment will be considered with a MMSE ≤ 24, and/or suffering from dementia, Alzheimer's disease, or a related mild-to-severe cognitive impairment, and/or a unability to follow simple indications through the ORAS form. The participant may live in a protected unit of the nursing home. Participant with a wheelchair, as long as a physical recovery can be expected, can be included in group 4. The objective of group 4 is to prevent disorientation and risk factors for falls by improving general motor capacities.

**Figure 2:**
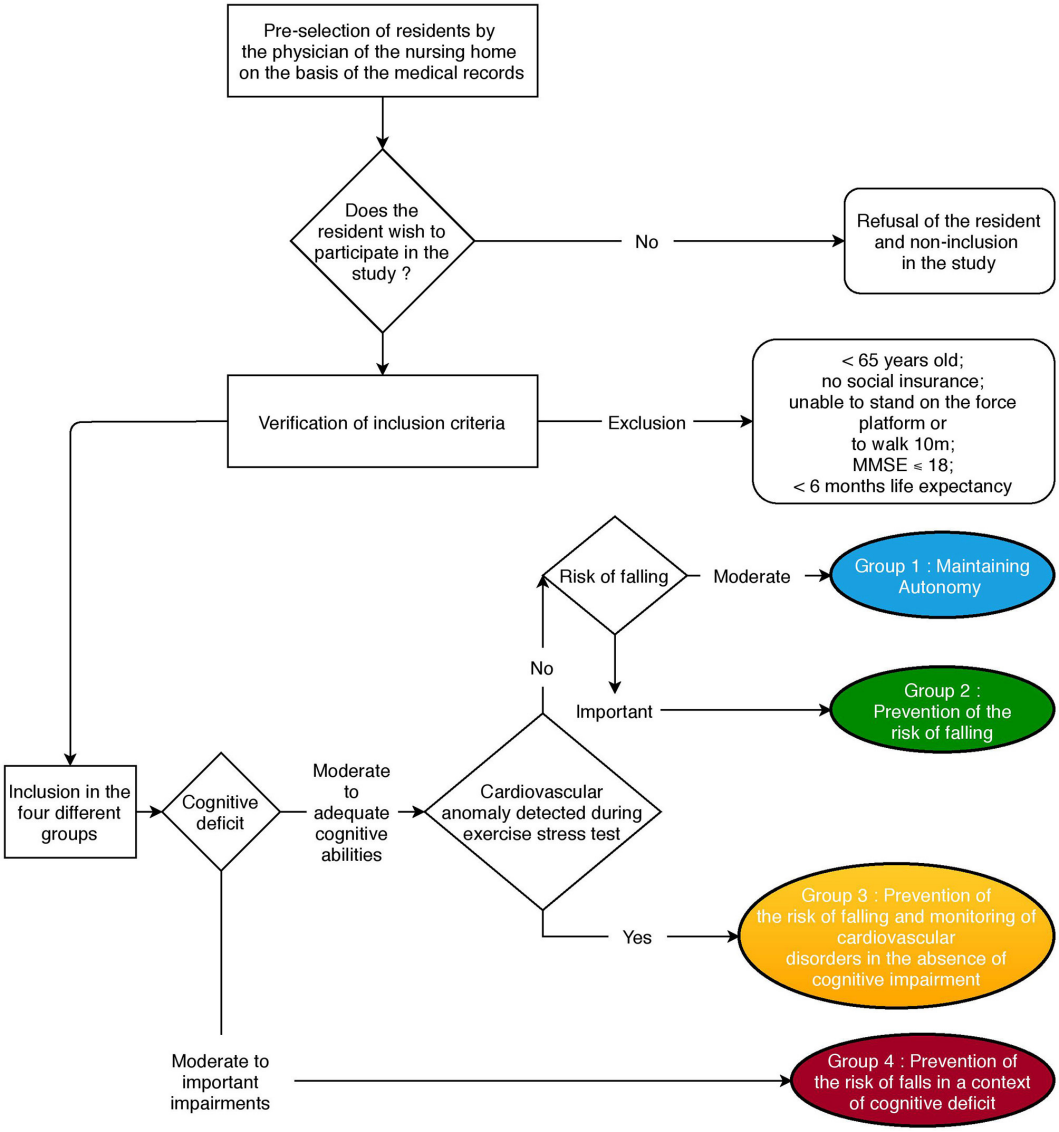
Flowchart of the distribution of residents in the different groups of APA after checking the adequacy of the selection criteria.

To summarize the allocation of participants in each group, from the list proposed by the coordinating physician of the nursing home, people are informed about the implementation of the clinical trial in their residence. They obtain the information note and are informed about the study by the clinical trial investigator. They are then assessed using the ORAS form to look for cognitive deficits that will lead to their inclusion in group 4. If the cognitive deficits are limited, the screening for cardiovascular anomalies allows them to be referred to group 3 if necessary. If there are no cardiovascular anomalies during the stress test, and depending on their risk of falling as determined via the ORAS form, participants are allocated to either group 2 or group 1, if their risk of falling is considered high or moderate, respectively (Figure 2).

#### 2.3.4. Participant Timeline

Following the opening visit of the residences and their inclusion in the clinical trial, the residents likely to be included in the study are listed by the coordinating physician of the center. The investigator then meets with the listed residents to ensure that the inclusion criteria are respected. After information is provided and written informed consent is obtained, the investigator also collects the baseline data necessary to follow the resident for the next 6 months (Table 1). For the 6 months, the static and dynamic balance of the residents will be recorded monthly, while a daily measurement of falls and their circumstances is provided by the health care team. This procedure should reduce the risk of recall bias that is often present with self-reporting of the number of falls by participants. After these 3-month follow-up without APA, the setting up of APA sessions allows to propose a program adapted to the capacities of the selected residents. The choice of an APA program is based upon the most representative participants, i.e., those who are more likely to form a homogeneous group. Over the next 3 months, 24 exercise sessions will be followed by the selected participants, twice a week for 1 h.

**Table 1:** Measurements time lines.

	**Inclusion**	**Monthly measures**	**APA programs**	**Final measurement**
	**D0**	**D1 to D90**	**D90 to D180**	**D180**
Informed consent (R)	✓	-	-	-
Determination of GIR (S)	✓	-	-	✓
Risk factors evaluation (R)	✓	-	-	✓
MMSE (S)	✓	-	-	✓
Pain assessment (S)	✓	-	-	✓
Functional balance assessment (BBS or MMT) (S)	✓	-	-	✓
Instrumental balance assessment (R)	✓	✓	✓	✓
Evaluation of residents participation during APA groups (R)	-	-	✓	-

*The different measures and information recordings take place at the period where the check marks (✓) are present for every included resident. The (S) represents standard follow-up care and (R) represents research purpose as an additional procedure for the follow-up of included residents*.

The observation period without APA will allow to define a baseline for all participants while the APA programs will be used to test the sensitivity of our predictive models in the improvement of the risk of falling. After these 6 months of follow-up (3 months without APA and 3 months with APA), the residents return to their daily activities and a new cohort is selected within the residence for the next 6-month period.

If participants are already benefiting from physiotherapy or other therapies to reduce the risk of falling, we do not intend to stop other treatments. This choice is explained by the objective of the study, which is not to compare the effects of physical activity between groups but to validate the relevance of predictive markers in relation to the evolution of the risk of falling assessed by clinical evaluation and the number of falls incurred.

#### 2.3.5. Physical Activity Programs

Sessions are organized in groups of 5–10 participants. Each session supervisor (physiotherapist or APA teacher) is in charge of organizing two sessions per week and per group. The sessions last from 45 min to 1 h with at least 20–25 min of effective exercise, the rest of the time being devoted to the installation of the participants and rest periods (Additional File 3). Outside the sessions, some exercises can be carried out in the resident's room, if they have been precisely explained and if the resident do them safely during the group sessions. Compression stockings or walking aids must be kept during the session and verification by a physiotherapist is encouraged.

These four multidimensional programs are composed of exercises that follow the ProFaNe classification, i.e., physical exercises for balance, coordination, endurance, muscular strengthening, three-dimensional exploration, overall physical activity, and flexibility (81). These programs are based on specific training identified as effective in reducing the risk of falling (51, 82). They gather static balance exercises, such as modification of the base of support, and dynamic balance exercises with upper limb–lower limb coordination, exercises focused on trunk motion, functional tasks with the displacement of objects in the three dimensional space, lower limb muscle strengthening, and gait exercises with obstacles (such as mats or foam for example). The training of new motor synergies will allow the restoration of compensation mechanisms, which help to stabilize in case of internal or external disturbance. The training will also encompass sit–stand–walk transfers and optimization of attentional resources (such as multitask activities). Examples of group sessions are presented in the Supplementary Material (Additional File 3).

Once all participants have settled in and the objectives have been explained, the session begins with warm-up exercises of low difficulty on a chair. The session continues with exercises sitting or standing in front of a chair. Then, a rest period is established to restore the cardiovascular and respiratory capacities of the participants. This is also the opportunity to make the participants aware of their physiological capacities through their breathing or heart rate. After a short rest period, the session continues with ball exercises, target games, or any other exercise to be done in a group to federate the participants and energize the session. The exercises are chosen from the catalog provided to practitioners, according to the theme of the session and the position required (Additional File 3) in order to limit the number of transfers. Indeed, changing positions too often could be particularly tiring for the participants and be detrimental to a targeted work. Physical exercises, especially those that require balance, must always be done safely. Warning notes and possible modification of the exercise are provided in the exercises sheets.

It will sometimes be necessary to adapt the exercise, its duration, or the number of repetitions in relation to the capacities of the participants on an individual basis. The progression of the exercises is done by increasing the number of series (from 1 × 10 repetitions to 2 × 10 repetitions for example), the weight of the resistances, the duration in challenging positions, by reducing the support surface, by working in the 3 dimensions of space or by increasing the height of the obstacles or the distance from the targets. Every 10 sessions, the difficulty should be revised.

## 3. Data Management and Statistical Analysis

Data will be collected by the study investigator and by the coordinating physicians of the institutions. For details concerning falls, the coordinating investigator will collect the standardized forms. For static balance analysis, a pre-processing algorithm is used to filter and resample the signal (83). An algorithm is used to verify the quality of the data from gait recording and automatically detect the steps from the IMU signals (60, 77, 78, 84). The computed parameters used to predict the risk of falling are presented in previous publications for both static (11, 12, 62) and dynamic balance assessment (35, 61, 85). In these studies, non-linear models (Ranking Forest and Gaussian Mixture Model) are used for classification. The validation of these descriptors obtained a good classification in retrospective fall recordings, which suggests that they will also be useful for the prospective classification of the risk of falling. The results will be considered significant at the 5% threshold.

In case of a flagrant behavioral abnormality (e.g., obsessive-compulsive disorder, psychiatric disorder, and so on), the participant's data will be excluded from the study. Missing, unused, or invalid data will not be included in the results or published. All data are stored on protected servers with regular backups. Internal audits are planned to verify the reliability of the data. Given the nature of the study, the expected adverse events gather traumatic problems related to fall from the WBB, during gait or physical activity.

### 3.1. Sample Size Computation

In order to develop a predictive tool that quantify balance with sufficient sensitivity to the evolution of the risk of falling during a 6-month longitudinal follow-up, it is necessary to quantify the expected improvement of posturographic markers. Previous results have shown the feasibility to estimate the risk of falling with force plates (13, 86) or IMU during walking (31) but, to date, we have not been able to find a study that evaluates monthly and longitudinally the balance via these sensors for institutionalized elderly people. Hence, we used previously published data (87) to estimate the necessary sample size because of the similarity with this study. They attested of an evolution of the COP sway area after 3 months of physical activity. We use the following formula to calculate the number of subjects required (88):


(1) $$\begin{array}{l} N=\frac{\sigma^{2}\left(Z_{\beta }+Z_{\frac{alpha}{2}}\right)}{d^{2}} \end{array}$$

where

- *N* is the number of participants per group;
- σ^2^ is the variance of COP surface area from (87);
- *Z*_β_ is the normal standard variable for a 90% statistical power (*Z*_β_ = 1.28);
- $Z_{\frac{alpha}{2}}$ is the normal standard variable for a significant threshold stated at 95%, ($Z_{\frac{alpha}{2}}=1.96$);
- *d*, the difference between two means [*d* = 0.11*cm*^2^ from (87)].

Hence, 72 participants per group are required. We estimate a 20% risk of loss of follow-up for this type of prospective study with the elderly people (89, 90), mainly due to the mortality (3). It seems necessary to include a minimum of 348 participants in total.

### 3.2. Statistics

The main evaluation criterion is the predictive value of the indicators measured monthly, correlated with the daily recorded number of falls. The predictive value will be quantified by assessing the threshold of the predictive score out of 100 based on a sensitivity of at least 80% and maximum specificity (Youden's index). It will be computed from the receiver operating characteristic (ROC) curve and its area under the curve (AUC), measuring the classification between “high risk of falling” and “low risk of falling” (binary criterion) (91–94). This classification is subsequently checked using the fall record sheets fulfilled by the medical staff. This standardized fall recording should reduce the number of “silent events,” such as forgotten fall as it may appears with fall history questionnaires, and provide more qualitative data around the falls (79).

The predictive value will also be evaluated by measuring the positive predictive value and negative predictive value from the binary classification. We will also look for a correlation between biomechanical markers and the other recorded variables [clinical tests (BBS or MMT), pain, and independence (AGGIR)] as well as the severity of falls according to an adaptation of the Hopkins Falls Grading Scale: “Grade 1: Fall with no immediate physical consequences; Grade 2: Fall with minimal consequences that did not require medical assistance; Grade 3: Fall that required medical assistance but did not lead to hospitalization; Grade 4: Fall that led to hospitalization or death” (95).

The detection of a clinical change through an analysis of variance (ANOVA) will be calculated via the within-subject standard deviation, also called standard error of measurement (SEM). The SEM can be calculated from the square root of the mean square error term in an ANOVA and will be computed throughout the follow-up of the first 3 months, and then will allow to determine the minimal detectable change (MDC) (96, 97). The MDC is the minimum value to attest to a clinical evolution of the subject that cannot be explained by an error in the measurement. This should be more than 2.77 times the SEM of the fall risk score, if the variables are normally distributed. We will use an anchor approach to measure MDC, if it is not possible to measure the within-subject standard deviation (when participants move from one category to another—“low risk of falling” to “high risk of falling”—without being able to make repeated measurements due to hospitalization, for example). These methods correlate the change from a score (here the fall risk score) to an objective external evaluation (the number of falls) (98).

The acceptability of using the predictive score will be measured using the questionnaire (Additional File 3) based on the Technology Acceptance Model and the 7-point Likert scale (99).

## 4. Discussion

Physical activity, whether associated with other interventions or not, shows a significant decrease in the rate of falls resulting in injury (56). While the beneficial effect of physical exercise on the physiological capacities, even in highly dependent elderly people, has been highlighted in many studies (10, 44, 45, 100–102), the impact of APA programs on posturographic parameters seems to be a matter of debate (103). Nonetheless, it has previously been shown to improve postural stability with a reduction in mediolateral sway (104), total sway length, and mean sway velocity (37, 51). Improvements in the static balance can therefore be expected (105).

On the other hand, the way in which exercise programs are implemented can vary the expected benefits and too little publication presents in details the programs provided. Given this lack of information, the choice of the best methods of implementation, particularly in nursing homes, remains an open question (45). In the Postadychute-AG clinical trial, the choice was made to propose four physical activity programs according to the cognitive deficits, cardiovascular, and fall risks. Repeated measurements of static and dynamic balance, as well as the evaluation of risk factors at the beginning and end of inclusion, will serve to attest to the evolution of the individuals' capacities.

Johansson et al. showed that the WBB could be used to assess the risk of falling in elderly subjects (13). Indeed, beyond a certain threshold value of the COP displacement, the risk of falling is increased by 75% in community dwelling elderly people. Although this study gathered 1,877 participants, the authors were not able to implement regular measurements (only punctual measurements at 6 and 12 months). They highlight the lack of follow-up as a limit to extending their results to the clinical setting. These results are joined by those of other authors using WBB (86) or ILG during walking (31). In a follow-up of 130 participants, (106) observed significant differences in posturographic parameters between fallers and non-fallers based on retrospective data. A conclusion can therefore be drawn on the feasibility of a one-time measurement for assessing the risk of falling at 3, 6, and 12 months. However, the feasibility of periodic monitoring as a method of longitudinal fall risk assessment, combining COP characteristics and gait parameters, remains to be proven (107). In terms of expected outcomes, no studies appear to have studied MDC over several months of follow-up, especially in institutionalized seniors. However, in a previous study (108), the intra-session MDC was estimated to be ±1.2 and ±0.6 mm/s for the AP mean velocity and ML mean velocity variables, respectively. Both variables have very good between-day reliability (0.92 [0.87–0.97] and 0.91 [0.85–0.97], respectively, for the AP and ML directions) (109), which explain the discrimination capacities of these COP variables (108). Even though the variability in COP characteristics is likely to be higher between sessions, rather than within sessions, similar results can be expected.

Gait impairments can have a significant impact on quality of life and independence. In neurological disorders, gait analysis by IMU has shown interest in quantifying and monitoring gait degradation (63). A review of the literature has already highlighted the interest of several markers for distinguishing older fallers (110), such as step length, gait speed, stride length, and contact time variability. From this list, and other variables especially in the U-turn (111), the developed algorithms will be able to provide a risk score combining static and dynamic measures. On the basis of previous results (112), it is likely that the analysis of temporo-spatial variables of gait can effectively distinguish fallers from non-fallers in elderly institutionalized population. The SEM and the MDC of gait velocity were measured at 6.5 and 18.1% at their maximum, respectively, while the variability of the other gait parameters could be higher (113). Hence, markers derived from gait velocity could be sensitive enough to change over repeated measurement in medium-term.

The goal of using a standardized assessment of falls and cardiovascular risks and cognitive deficits, through the ORAS form, is to evaluate risk factors, on the one hand, and to propose a physical activity programme that can be performed by a homogeneous group, on the other hand. All the four programs focus on mobility and physical improvement. However, in order to set up a series of exercises adapted to the deficits, it is necessary to take into account the potential pitfalls inherent to the difficulty of the physical activity as well as the participants capacity of understanding the supervisor's recommendations. It is indeed necessary to take into account the undesirable effects due to physical activity to attest its effectiveness. A recent meta-analysis was unable to find any adverse effects significantly associated with APA, not even muscle soreness (56). There is a lack of reporting adverse effects in prevention programs for older adults (114) and, at the same time, individual clinical trials do not collect sufficient data to study the adverse effects of these programs, which have a low rate of occurrence. In another meta-analysis, no adverse effects were found in any of the 30 included studies (115). Nevertheless, it is important to be vigilant about the prescribed exercise dose or the instructions provided to ensure rest periods and avoid overtraining.

Considering the difficulty of identifying a single cause of falling (116), rather than proposing exercises focused on an identified cause of falls, the creation of the groups should be guided by the possibility of following the supervisor's instructions from the participants, so that people in the same group are able to perform the exercises. The benefits of physical activity in the elderly are global, i.e., improving many functions from balance to gait quality, including cardiovascular, respiratory, and possibly cognitive functions (117–121). Previous results have shown benefits in favor of programs that add in-depth geriatric risk analysis and subgrouping according to individual abilities, especially concerning participants' cognitive impairment (MMSE ≤ 19) (57). The results of trials on the prevention of falls in cognitively intact elderly people are nevertheless difficult to generalize to people with dementia given the difficulty of following the session supervisor's instructions (122). It is then appropriate to propose a group with important cognitive impairments and separate them from persons with preserved functions. For cardiovascular disorders, physical exercise could constitute a risk of adverse event, a factor limiting the benefits of APA and ultimately lead to a disengagement of the participants. Moreover, the correlation between the proposed exercises and the benefits on the different functions remains tenuous in the absence of more data among people living in nursing homes (45), which makes it necessary to tailor the programs according to criteria that seek to homogenize the groups.

## 5. Conclusion

To counter the limitations of clinical assessments, postural parameters and automated gait analysis could be effective in providing a quantification of balancing capabilities. They would provide a better understanding of the physiological mechanisms impacted by aging (123). In addition, distinguishing elderly people at risk of falling, or predicting future falls during longitudinal follow-up, has been shown to be effective with classic stabilometric parameters (11, 14, 124). At the same time, automated gait analysis allows the extraction of biomechanical parameters to facilitate early diagnosis of future fallers (125, 126). However, too few studies follow elderly people longitudinally with repeated measurements to observe posture degradation. The lack of data is even more striking among institutionalized individuals who, however, would benefit most from being monitored over several months. This lack of information on the usefulness of posturography to follow these people and allow a fine and sensitive evaluation of the risk of falling is the problem that this clinical trial aims to solve. In addition to providing information during a follow-up of institutionalized persons twice 3 months concerning their balance capacities, the implementation of 4 programs of APA could make it possible to evaluate the benefit of such programs on the number of falls as well as on the static and dynamic balance parameters.

## Author Contributions

All author have made substantial contributions to the conception and design of the work. The acquisition, analysis, and interpretation of data will be made by FQ. FQ, P-PV, DR, FB-H, and PZ have drafted the work and revised it. All authors have approved the submitted version. P-PV, DR, FB-H, PZ, and ML carried out management and financing activities for the research project.

## Conflict of Interest

The authors declare that the research was conducted in the absence of any commercial or financial relationships that could be construed as a potential conflict of interest.

## Supplementary Material

Additional File 1 Oras form, for cognitive, cardiac, and fall risks assessment. The evaluation form is in .docx format to be easily read and printed by health care teams. The different elements of the form are to be filled in by the doctor and a rehabilitation worker at least. It is estimated that it will take 30 min to complete, but it is advisable to fill it in several times and with a multidisciplinary approach.

Additional File 2 Exercise sheet. Presents the exercise and associated comments. The title and descriptions provide information on the action to be performed, while contextual information (such as position or difficulty) allows the facilitator to organize the exercise.

Additional File 3 Group session description. Presents the order of exercises during a typical session. The number of repetitions is for information only, so that the speaker can adapt the session according to the theme he or she wishes to work on.

Additional File 4 Fall score usefulness evaluation form. Evaluation of the Cognac-G measurement method by the nursing staff. “Perceived usefulness” and “Perceived ease of use” are assessed with 10 items each. “Intent to use” is assessed with 4 items.

## Abbreviations

AGGIR: Autonomy Gerontology Group Iso Resources
ANOVA: analysis of variance
ANSM: Agence nationale de sécurité du médicament
ANRT: Association Nationale de la Recherche et de la Technologie (National Association for Research and Technology)
APA: adapted physical activity
BBS: Berg Balance Scale
CNIL: Commission nationale de l'informatique et des libertés (National Commission for Informatics and Liberties)
COP: center of pressure
CPP: Comité de Protection des Personnes (Protection of Persons Committee)
IMU: inertial measurement unit
MMSE: Mini Mental State Examination
MMT: Mini Motor Test
MDC: minimum clinically detectable change
ORAS: ORPEA Risk Assessment Sheet
ProFaNE: Prevention of falls network Europe
SEM: standard error of measurement
WBB: Wii Balance Board.
