# Supplementary material for: Postadychute-AG, Detection, and Prevention of the Risk of Falling Among Elderly People in Nursing Homes: Protocol of a Multicentre and Prospective Intervention Study
Source: Front Digit Health. 2021 Jan 27;2:604552. doi: 10.3389/fdgth.2020.604552 (PMC8521935; doi:10.3389/fdgth.2020.604552)
Supplement: Additional File 1 — Oras form, for cognitive, cardiac, and fall risks assessment. The evaluation form is in .docx format to be easily read and printed by health care teams. The different elements of the form are to be filled in by the doctor and a rehabilitation worker at least. It is estimated that it will take 30 min to complete, but it is advisable to fill it in several times and with a multidisciplinary approach. [file Data_Sheet_1.PDF]

ORPEA Risk Assessment Sheet -ORAS- for assignment in APA programs

Assessment of the elderly person's fall and cardiovascular risks for inclusion in an Adapted Physical Activity program.

The purpose of this assessment is to evaluate the cardiovascular, cognitive and balance capacities of residents who may be included in Adapted Physical Activity programs. This form must be completed by a trained caregiver (doctor, physiotherapist, nurse) and then validated during a multidisciplinary meeting. The boxed sections should be filled in by a doctor and/or with the medical record, the rest can be filled in by a rehabilitation therapist. It must be indicated whether the information obtained comes directly from a resident, a relative or the health care staff. A stopwatch, a grip-test and a saturometer are necessary to carry out this assessment. Fill in the form visibly with a blue pen

Evaluation carried out in: _ _ _ sessions

Healthcare professionals: Name, position: _ _ _ _ _ _ _ _ _ _ _ _ _ _ _ _

Date: _ _ _/_ _ _ /_ _ _ Name, position: _ _ _ _ _ _ _ _ _ _ _ _ _ _ _ _

Resident:

Ms. ☐ Mr. ☐ Name: _ _ _ _ _ _ _ _ _ _ _ _ _ First name: _ _ _ _ _ _ _ _ _ _ _ _ _
Birth date: _ _ _/_ _ _/_ _ _

Number of known falls in the last 12 months: _ _ _ _ _

☐ Post-Fall Syndrome ☐ Phobia of standing upright
☐ Does not exhibit psychomotor dysfunction ☐ No known fall

- **Neuropsychiatric index –NPI-: _____**

Behavioral disorders found:
☐ type Non-Productif
☐ type Productif
☐ no behavioral disorder

- **Cardiovascular and Pulmonary Assessment:**

All institutionalized elderly people are at risk, a priori. The presence of a clinical symptom of recent onset, chest pain or dyspnea for low effort should alert. For risk factors: active smoking, unbalanced diabetes, unbalanced hypertension, recent arrhythmia attacks should be a warning sign.

- Simple technical elements (collected by the physiotherapist or physician)

☐ Saturation below 95%

- Cardiac and/or respiratory symptoms (questioning)

☐ Chest pain for low effort
☐ Chest pain at rest de repos
☐ Dyspnea for low effort
☐ Palpitations
☐ Unclean expectorations
☐ Cough
☐ No risk factor

- Simple clinical findings (observation)

☐ Cyanosis of the lips / extremities
☐ Irregular pulse
☐ Ventilation pauses
☐ Abnormal respiratory rate (normal between 12 and 20 cycles/min)
☐ No risk factor

- Known cardiovascular risk factors (to be completed by a physician or retrieved from medical records)

☐ BMI > 30 kg/m²
☐ Notion of excess cholesterol treated or not treated
☐ Diabetes with or without insulin
☐ Active or stopped (but important) smoking
☐ Sleep apnea (with or without device)
☐ Stress (anxious profile, notion of treatment)
☐ Hypertension (treated or untreated)
☐ Heredity
☐ No risk factor

- Known cardiovascular or pulmonary history (increased risk, to be completed by a physician)

☐ Pulmonary arterial hypertension
☐ Pulmonary fibrosis
☐ Pulmonary thoracic surgery (whatever the cause)
☐ known COPD
☐ Oxygen therapy
☐ Heart rhythm disorders
☐ Known coronary artery disease (stent, bypass surgery, heart attack)
☐ Known valvulopathy with or without surgery
☐ Arteritis of the lower limbs
☐ Carotid stenosis (operated or not)
☐ Aortic aneurysm surgery
☐ Regressive stroke or TIA (transient ischemic attack)
☐ Heart Failure
☐ EKG abnormality
☐ No previous history

- **Elements for increasing the risk of falls (first part):**
  To be completed by the physician or with the medical file:

☐Temporo-spatial disorientation
☐ Sequelae of peripheral neurological disorders
☐ Dizziness
☐ Cardiovascular problems (hypotension, arrhythmia ...)
☐ Stroke sequelae
☐ Alcohol consumption
☐ Extra Pyramidal Syndrome
☐ Uncorrected visual deficit
☐ Urinary disorders
☐ Hearing disorders
☐Cognitive impairment: MMSE < 24
☐ Risk of drug iatrogenicity
☐No additional factor

Inclusion in the program :

☐ **Inclusion authorized after assessment by the physiotherapist**

Non-inclusion in the program if:

☐ Medical Contraindication for inclusion in the program (cardiac, respiratory or other instability) → *Termination of the assessment and definitive non-inclusion of the resident*

- **Test of effort when walking or on a hand-held cyclo-ergometer:**

Walking test

☐ performed for 3 minutes with verbal stimulation

☐ prolonged up to 6 minutes with verbal stimulation

Or

Hand-held cycle ergometer test at 60 rpm

☐ started at 20 Watts with a 10W increment every minute for 3 minutes

☐ prolonged up to 6 minutes with increment

- Pulse ox < 90% or rapid drop
- Heart rate = rest + 30%
- Blood pressure > 180 (systolic)/110 (diastolic)
- Stopping in the middle of the session: _

☐ Contraindications to the APA program if any of these factors are present → Additional specialist advice (cardio, pneumo...) required

☐ **No particular problems, continue with the evaluation**

The rest of the assessment can be carried out by a physiotherapist if there are no contraindications.

- **Pain assessment (interrogation):**

- Location: _ → VAS quotation: _ _ /10

☐ Impossible: _

- **Fragility of the resident (questioning):**

Information provided by: ☐ the resident ☐ a relative ☐ healthcare professional

- Can you walk on flat ground?

☐ Without technical support ☐ With technical support

☐ **With human assistance** ☐ **No**

How many steps or meters: _

- Can you walk up and down the stairs?

☐ Without technical support ☐ With technical support
☐ With human assistance **☐ No**
How many de steps/ floors: _________________________________

- Can you walk outside of the building?

☐ Without technical support ☐ With technical support
☐ With human assistance **☐ No**
How far: ____

- Can you walk more than 200 meters?

☐ Without technical support ☐ With technical support
☐ With human assistance **☐ No**

- When was the last time you did these activities? _________________

- **Balancing Capacities:**

Maintaining the sitting position:

☐ Without technical support
☐ With technical support
☐ Impossible : __________

Maintaining standing upright:

☐ Without technical support
☐ With technical support
☐ Impossible : __________

Initial evaluation of the risk of falling:

☐ Risk of falling to be weighted by the following paragraph
☐ High risk: 1 factor in bold from the two previous sections

- **Elements for increasing the risk of falls (second part)**

Asked the resident to sit on a chair, then stand up, stand on one foot and then walk three meters before returning to sit. Check the items below if the person has low performance and therefore an increased risk of falling.

☐ Sitting instability
☐ Unipodal station < 5 seconds
☐ Upright-sit transfer not possible
☐ One foot does not exceed the other in stance phase when walking.
☐ Timed up and go test >20 seconds with or without technical assistance

☐ Rises very slowly or with help
☐ Orthostatic hypotension
☐ Slow walk < 1 m/s
☐ Improper use of walking aids
☐ No additional factors

Perform an orthopedic assessment of the resident in a sitting or lying position.

☐ Functional orthopedic disorders:

- Rachis:_ _ _ _ _ _ _ _ _
- UL:_ _ _ _ _ _ _ _ _ _ _
- LL:_ _ _ _ _ _ _ _ _ _ _

☐ Skin and trophic disorders of the lower limbs

☐ No orthopedic disorder

☐ Problem of supports/foot (footwear, deformation, deep sensitivity)

☐ Sarcopenia (Grip Test: ♂ < 26 kg, ♀ < 16 kg)

Conclusion of the intermediate assessment of the risk of falling:

☐ Moderate risk

☐ High risk: ≥ 3 additional factors in first and second parts of the Elements for increasing the risk of falls

- **Cognitive abilities:**

- Point to the "in front of you":	Yes	☐	No	☐
- Point to the "behind you."	Yes	☐	No	☐
- Touch his left knee with his right hand:	Yes	☐	No	☐
- Touch his right knee with his left hand:	Yes	☐	No	☐
- Show the left ear with the right index finger:	Yes	☐	No	☐
- Possibility of double-tasking (walking + talking):	Yes	☐	No	☐

CONCLUSION:

0 -- -- -- -- -- -- -- -- -- 3 -- -- -- -- -- -- -- -- -- 6

☐ **Insufficient abilities** ☐ **Moderate** ☐ **Sufficient**

- **Assessment of motivation:**

☐ Fanatic (at risk)

☐Motivated (sometimes insufficient)

☐ Do the minimum (at the very least)

☐ In rebellion (nuisance to the group)

☐ Involved (ideal)

☐ Stimulated (requires stimuli)

☐ Demotivated (do nothing and risk of contagion)

Inclusion in the program:
☐ Group 1 ☐ Group 2 ☐ Group 3 ☐ Group 4
☐ Further examination required for inclusion in a program

Contraindications to participating in APA programs:

1/ definitive: _

2/ to be reevaluated in_ _ _ _ _ _ week(s) _

Profile of patients expected in each group:

- **Group 1 Maintenance of Autonomy: "Group 1 residents do not have a cardiovascular disorder, possibly some cognitive deficits, but do not reside in a protected unit. They are autonomous in their rooms, moving spontaneously and regularly within the residence. Patients in wheelchairs cannot be integrated into this group. »**

- **Group 2 Fall Risk Prevention: "Group 2 residents do not have cardiovascular problems, possibly some cognitive problems, but can finally follow the indications of the cognitive assessment on the form. A group 2 resident moves alone in his room but presents a significant risk of falls with a history of falls in the last 6 months. Patients in wheelchairs can be included in this group if they are able to make transfers and recovery of physical abilities is possible. »**

- **Group 3 Falls risk prevention and cardiovascular monitoring in the absence of cognitive impairment: "Group 3 residents have had a recent, currently stabilized cardiovascular event that may warrant exercise reconditioning. These cardiovascular disorders may be the cause of falls. If cognitive problems are present, they do not prevent group activity sessions. The wheelchair is not a contraindication to inclusion in this group."**

- **Group 4 Prevention of the risk of falls and disorientation in a context of cognitive impairment: "Group 4 residents present moderate to severe cognitive impairment, or have Alzheimer's disease or a related disease at a stage of the disease requiring special attention (inclusion in a protected unit, MMSE ≤ 24, etc.) but allowing for follow-up sessions in group 4. For the resident, his cognitive problems have an impact on his daily life and he must be supervised for his activities. Wheelchair patients with cognitive impairment in the foreground can join this group."**

Comments: _
_ _
