# Supplementary material for: Postadychute-AG, Detection, and Prevention of the Risk of Falling Among Elderly People in Nursing Homes: Protocol of a Multicentre and Prospective Intervention Study
Source: Front Digit Health. 2021 Jan 27;2:604552. doi: 10.3389/fdgth.2020.604552 (PMC8521935; doi:10.3389/fdgth.2020.604552)
Supplement: Additional File 2 — Exercise sheet. Presents the exercise and associated comments. The title and descriptions provide information on the action to be performed, while contextual information (such as position or difficulty) allows the facilitator to organize the exercise. [file Data_Sheet_2.PDF]

Information to be taken into account before

Title of the exercise

Postadychute-AG, exercises program for the prevention of institutionalized older peo
risk of falling |

For which of the 4 groups this exercise is recommended

Forward projection of the centre of gravity

Comments and warnings	Group	Thematic
This exercise can be used as a method for assessing balance. Measure the increase in the distance of the fingers from the upright position to the bent forward position.	- Group 1	Balance

Thematic of the exercise to be in line with the "ProFaNe" theme of the session

Difficulty:

1 2 3 4 5 6 7 8 9

STANDING

Position during the exercise

Description
Try to do a series of long, slow forward bends, keeping your balance for a few seconds between each movement, with your arms outstretched.
**Adjustment of the exercise**
↑ Bring your feet together until your feet are touching and then bend forward with your arms outstretched.

Level of difficulty set a priori

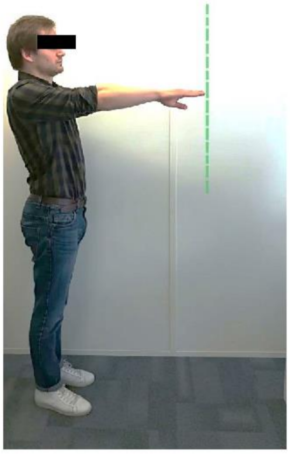

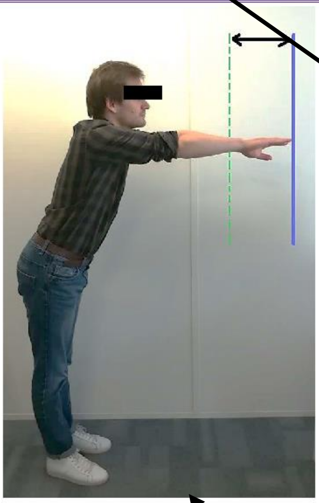

Example of adapting the exercise to the participant's abilities. An upward arrow indicates an adaptation to increase the difficulty. An arrow down indicates a decrease in difficulty.

Illustration of the exercise and possibly of the different steps
