# Supplementary material for: Postadychute-AG, Detection, and Prevention of the Risk of Falling Among Elderly People in Nursing Homes: Protocol of a Multicentre and Prospective Intervention Study
Source: Front Digit Health. 2021 Jan 27;2:604552. doi: 10.3389/fdgth.2020.604552 (PMC8521935; doi:10.3389/fdgth.2020.604552)
Supplement: Additional File 3 — Group session description. Presents the order of exercises during a typical session. The number of repetitions is for information only, so that the speaker can adapt the session according to the theme he or she wishes to work on. [file Data_Sheet_3.PDF]

First session group 1:

Installation		
**Description**		**Duration**
This example fits into the framework of group 1, for a "Coordination" theme. The aim of the exercises is to work on balance and the precision of gestures in a functional context. The exploration of three-dimensional space is at the heart of the session.<br>Residents sit on chairs arranged in a circle.		10 minutes
Warm-Up		
**Exercices**	**Progression**	**Duration**
☒ Upper Limbs Warm-up	10 repetitions without weights	5 minutes
☒ Lower Limbs Warm-up	10 repetitions without weights	
☒ Sitting thoracic extension	10 repetitions without weights	
**Exercices**	**Progression**	**Duration**
☒ Sit-stand transfer	With the help of hands	15 minutes
☒ Unipodal support	Fingertip support<br>(5 minutes)	
☒ Ball Throwing	Throwing with different types of passes (5 minutes)	
Pause		
**Description**		**Duration**
☒ Active recovery with standing chest extension		5 minutes
**Exercices**	**Progression**	**Duration**
☒Stepper	Stepper coordination exercise (left hand - right leg)	10 minutes
Return to calm		
**Description**		**Duration**
Participants resettle in their place and collect their belongings before leaving.		5 minutes

The exercises will become more complex with the number of repetitions and the axes of exploration (mediolateral, then anteroposterior and finally vertical).
