# Supplementary material for: Postadychute-AG, Detection, and Prevention of the Risk of Falling Among Elderly People in Nursing Homes: Protocol of a Multicentre and Prospective Intervention Study
Source: Front Digit Health. 2021 Jan 27;2:604552. doi: 10.3389/fdgth.2020.604552 (PMC8521935; doi:10.3389/fdgth.2020.604552)
Supplement: Additional File 4 — Fall score usefulness evaluation form. Evaluation of the Cognac-G measurement method by the nursing staff. “Perceived usefulness” and “Perceived ease of use” are assessed with 10 items each. “Intent to use” is assessed with 4 items. [file Data_Sheet_4.PDF]

Evaluation of the Centre Borelli's balance measurement method

by health care personnel

1. Perceived usefulness:

1.1. Using the balance score improves the quality of your work

Strongly disagree **Strongly agree**

1	2	3	4	5	6	7

1.2. Using the balance score gives you more control over your work

1	2	3	4	5	6	7

1.3. The balance score allows you to do your work faster

1	2	3	4	5	6	7

1.4. The balance score helps you in the difficult aspects of your work

1	2	3	4	5	6	7

1.5. Using the balance score increases your productivity

1	2	3	4	5	6	7

1.6. Using the balance score increases your work performance

1	2	3	4	5	6	7

1.7. The balance score is a useful means of monitoring a patient's balance

1	2	3	4	5	6	7

1.8. Using the balance score improves your work efficiency

1	2	3	4	5	6	7

1.9. Using the balance score is very useful in your work

1	2	3	4	5	6	7

1.10. In general, you find the balance score helpful in your work

1	2	3	4	5	6	7

2. Perceived ease of use:

2.1. You find it burdensome to use the balance score

Strongly disagree **Strongly agree**

1	2	3	4	5	6	7

2.2. Learning to use the balance score seems easy to you

1	2	3	4	5	6	7

2.3. Using the equilibrium score is often frustrating

1	2	3	4	5	6	7

2.4. You find it easy to use the balance score to get what you want (e.g., an assessment of the patient's balancing abilities)

1	2	3	4	5	6	7

2.5. You find that the balance score is too strict or not flexible enough

1	2	3	4	5	6	7

2.6. It's easy for you to remember how to use the balance score

1	2	3	4	5	6	7

2.7. Using the balance score requires a lot of mental effort

1	2	3	4	5	6	7

2.8. Using the balance score is clear and understandable

1	2	3	4	5	6	7

2.9. You find that exploiting all the variables of the balance score requires a lot of effort

1	2	3	4	5	6	7

2.10. In general, I find the balance score easy to use

1	2	3	4	5	6	7

3. Intent of use:

You would recommend the use of the balance score to your colleagues

Strongly disagree **Strongly agree**

1	2	3	4	5	6	7

3.1. You wish to use the balance score as often as possible during the period of availability

1	2	3	4	5	6	7

3.2. You intend to use all the variables of the balance score

1	2	3	4	5	6	7

3.3. You expect to use every week (at least once a week) the balance score

1	2	3	4	5	6	7
